# Use of Animal Models for Investigating Cardioprotective Roles of SGLT2 Inhibitors

**DOI:** 10.1007/s12265-023-10379-5

**Published:** 2023-04-13

**Authors:** Najlaa A. Al Thani, Maram Hasan, Huseyin C. Yalcin

**Affiliations:** 1 Research and Development Department, Barzan Holdings, P. O. Box 7178, Doha, Qatar; 2 Biomedical Research Center, Qatar University, P. O. Box 2713, Doha, Qatar; 3 Department of Biomedical Science, College of Health Sciences, QU Health, Qatar University, P. O. Box 2713, Doha, Qatar

**Keywords:** SLGT2, Diabetes, Heart failure, Cardioprotection, Gliflozins, Empagliflozin, Canagliflozin, Animal models

## Abstract

Sodium-glucose co-transporter 2 (SGLT2) inhibitors represent one type of new-generation type 2 diabetes (T2DM) drug treatment. The mechanism of action of an SGLT2 inhibitor (SGLT2i) in treating T2DM depends on lowering blood glucose levels effectively via increasing the glomerular excretion of glucose. A good number of randomized clinical trials revealed that SGLT2is significantly prevented heart failure (HF) and cardiovascular death in T2DM patients. Despite ongoing clinical trials in HF patients without T2DM, there have been a limited number of translational studies on the cardioprotective properties of SGLT2is. As the cellular mechanism behind the cardiac benefits of SGLT2is is still to be elucidated, animal models are used to better understand the pathways behind the cardioprotective mechanism of SGLT2i. In this review, we summarize the animal models constructed to study the cardioprotective mechanisms of SGLT2is to help deliver a more comprehensive understanding of the in vivo work that has been done in this field and to help select the most optimal animal model to use when studying the different cardioprotective effects of SGLT2is.

**Graphical Abstract:**

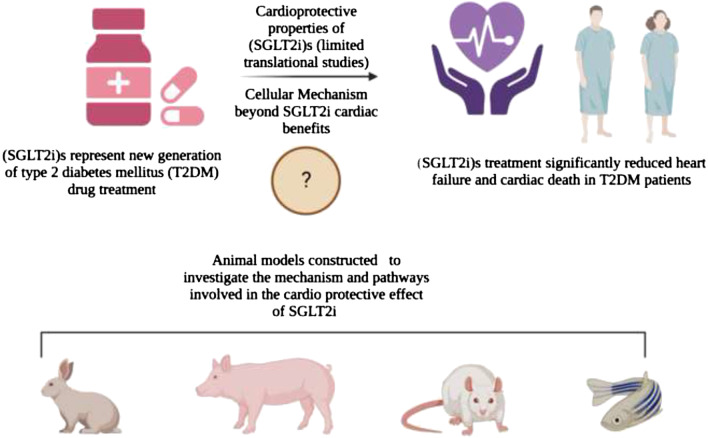

## Introduction

Sodium-glucose co-transporter 2 (SGLT2) inhibitors, also known as gliflozins, are a class of new-generation type 2 diabetes (T2DM) drug treatment. An SGLT2 inhibitor (SGLT2i), such as empagliflozin (EMPA), dapagliflozin (DAPA), and canagliflozin (CANA), effectively lowers blood glucose levels through an increase in glomerular excretion of glucose by the kidneys [1]. SGLT2is are different from insulin-based drugs which function by allowing glucose to be absorbed by the cell, for which in the long run, a resistance of uptake might be initiated [2].

**Fig. 1:**
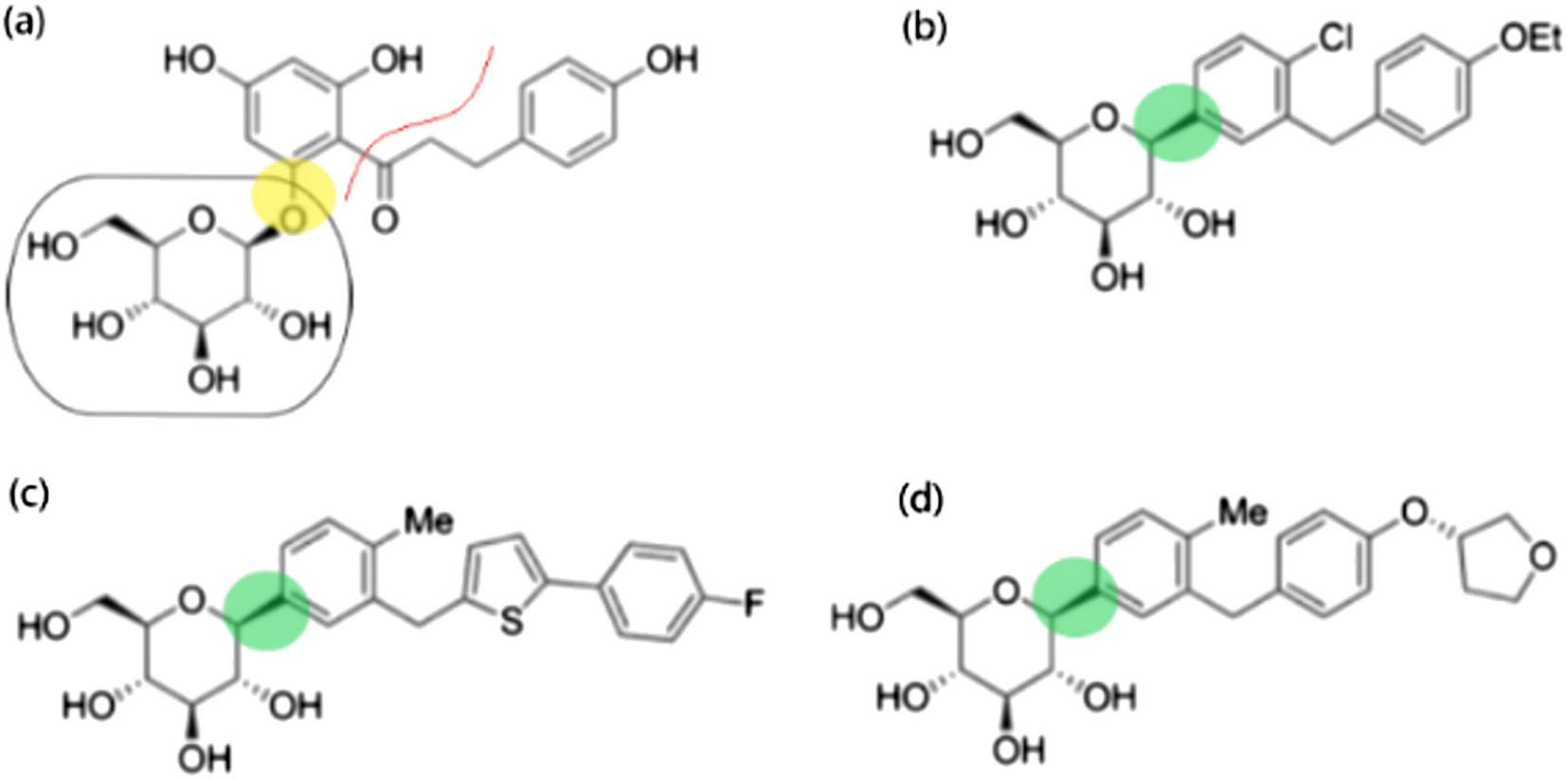
Chemical structure of (**a**) phlorizin with glucose moiety (circled) and aglycone tail (separated by red line); (**b**) dapagliflozin; (**c**) canagliflozin; (**d**) empagliflozin. O-C bonds (yellow) and C–C bonds (green) highlighted

**Fig. 2:**
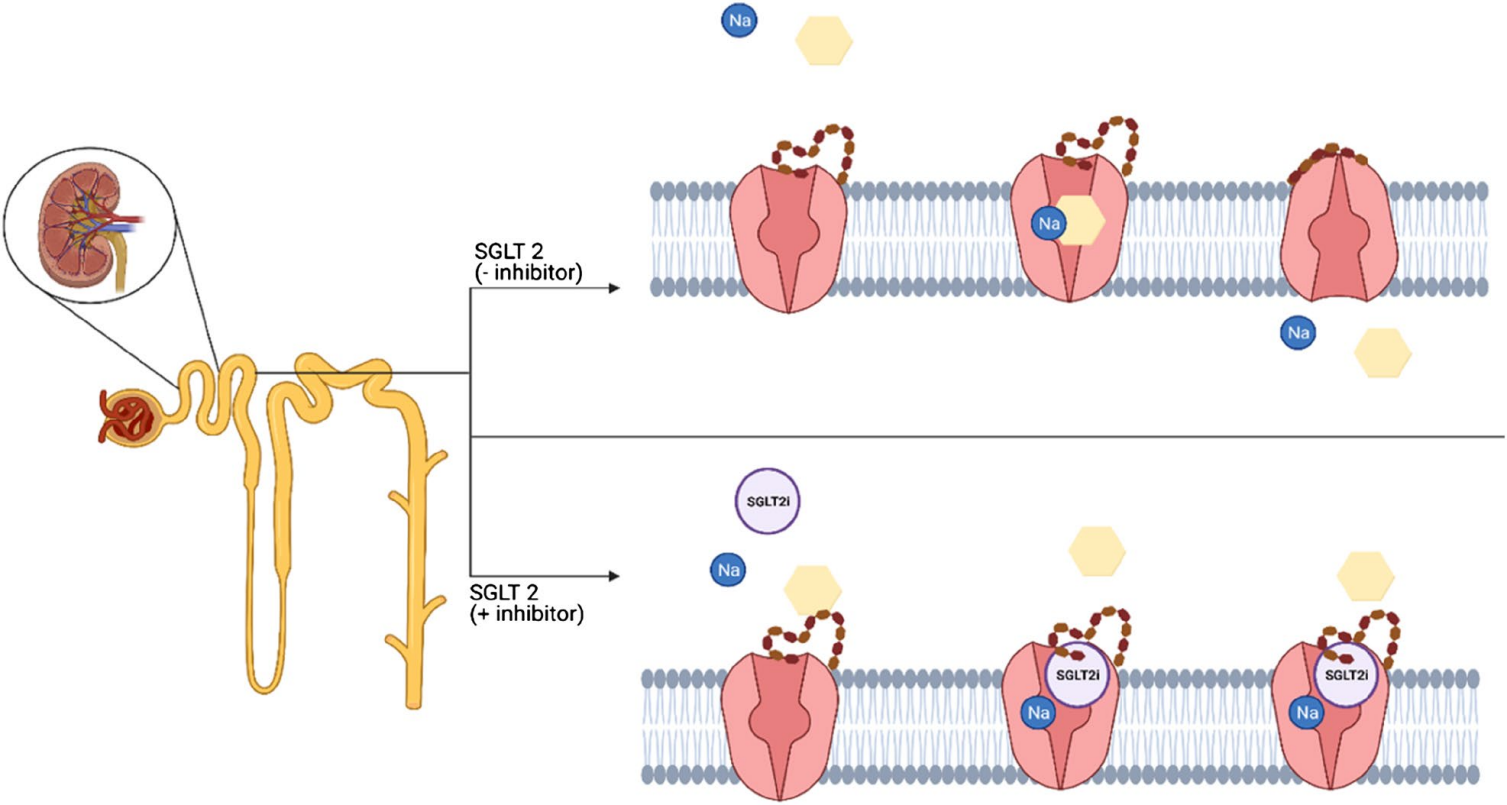
Allosteric model of inhibitor binding to SGLT2. Absence of inhibitor allows binding of [Na^+^] ion and glucose molecule (yellow). The binding of phlorizin-like inhibitor (SGLT2i) to the outward-facing state of SGLT2 leads to a partial closure of the outer transmembrane domain (red/brown peptide chain) in an induced fit mechanism. Created with Biorender.com

Pharmaceutical gliflozins have been modeled after the naturally occurring O-glycoside, phlorizin, which consists of a glucose moiety and aglycone tail attached to an aromatic ring. For all gliflozins, the glucose moiety is conserved and is bound to the aromatic ring via a C–C bond making it a C-glycoside and increasing its stability compared to phlorizin which utilizes an O-C bond. The aglycone tail differs for each gliflozin but all similarly utilize aromatic structures (Fig. 1) [3]. It has been proven that those key structural features of gliflozins enhance the cellular mechanism of inhibition via binding to SGLT2 transmembrane proteins located in the proximal tubule of the kidney. The glucose moiety competes directly with glucose through stacking interactions and hydrogen bonding to several sidechains within the SGLT2 binding pocket, whereas the aglycone tail increases the binding affinity and stability of gliflozins by exhibiting *π*–*π* stacking with aromatic elements in the SGLT2 binding pocket [4]. Binding of the an SGLT2i fixes SGLT2 in its outward facing conformation, which subsequently stops binding of glucose, preventing it from reentering the bloodstream (Fig. 2) [5]. SGLT2is have also demonstrated the reduced ability to bind, specifically, to SGLT1 transmembrane proteins that are found predominantly in the small intestine, kidney, and heart [6, 7]. The cellular mechanism behind the binding selectivity of an SGLT2i relies on the fact the SGLT1 binds two Na^+^ ions which compromise the induced fit mechanism of SGLT2is.

In addition to SGLT2is’ direct benefits in T2DM, studies have witnessed cardiac improvements in patients treated with gliflozins through large randomized clinical trials. These studies show the effect of SGLT2is primarily in T2DM patients at high risk of major adverse cardiovascular events (MACE) defined as cardiovascular death, myocardial infarction, or ischemic stroke which constitute as heart failure (HF) [8, 9]. The most notable major clinical SGLT2i trials include the EMPA Cardiovascular Outcome Event Trial in T2DM Patients (EMPA-REG OUTCOME) [10], the Canagliflozin Cardiovascular Assessment Study (CANVAS) program [11], the Dapagliflozin Effect on Cardiovascular Events–Thrombolysis in Myocardial Infarction 58 (DECLARE-TIMI58) trial [12], and the Dapagliflozin and Prevention of Adverse Outcomes in Heart Failure (DAPA-HF) trial [13]. The observed cardioprotective effects of these drugs have been summarized in the table (Table 1). EMPA-REG OUTCOME, CANVAS, and DECLARE-TIMI58 were able to correlate the relationship between SGLT2is and HF prevention in T2DM patients. However, the DAPA-HF trial [13, 14] explored the therapeutic potential of SGLT2is on HF patients, irrespective of the presence of T2DM, and found that DAPA significantly reduced cardiovascular mortality and hospitalization for HF. This suggests that the beneficial effects of SGLT2i on HF patients are not solely based on glycemic reduction and correcting metabolic disorder related to T2DM. The study highlights the following cardioprotective mechanisms that SGLT2i could be involved in decreasing blood pressure; ketone body metabolic activity; increase of red blood cells percentage and the hemoglobin level; and sympathetic nerve function and triggering of the enzyme activity of endothelial nitric oxide synthase [14]. Other hypotheses include whether cardioprotective mechanisms are mediated by SGLT2i binding directly to SGLT2 in cardiac cells or indirectly by upregulating SGLT1 expression in cardiovascular disease [7].

**Table 1:** Major clinical SGLT2i trials and their observed cardioprotective effects. Created with Biorender.com

Major clinical SGLT2i trial	SGLT2i used	Patient condition	Observed cardioprotective effect
Empagliflozin Cardiovascular Outcome Event Trial in T2DM Patients (EMPA-REG OUTCOME) [10]	EMPA	T2DM patients at risk of or have cardiovascular disease	Reduced the incidence of cardiovascular complications among T2DM patients, cardiovascular deaths, and hospitalizations for HF
Canagliflozin Cardiovascular Assessment Study (CANVAS) [11]	CANA	T2DM patients at risk of or have cardiovascular disease	Reduced the incidence of cardiovascular death, myocardial infarction and stroke among T2DM patients
Dapagliflozin Effect on Cardiovascular Events–Thrombolysis in Myocardial Infarction 58 (DECLARE-TIMI58) [12]	DAPA	T2DM patients at risk of or have cardiovascular disease	Reduction in cardiovascular deaths and hospitalizations for HF among T2DM patients
Dapagliflozin and Prevention of Adverse Outcomes in Heart Failure (DAPA-HF) [13]	DAPA	HF patients with reduced ejection fraction (HFrEF)	Reduction in cardiovascular deaths, HF events and also associated with an improvement in symptoms

In order to better understand the main mechanism behind SGLT2i’s cardioprotective properties, several animal models have been constructed. In this review, animal models used to study the cardioprotective mechanisms of SGLT2i will be summarized. We assessed the work that has been done with each animal model to help with the selection of most appropriate model when studying cardioprotective properties of SGLT2i. This paper aims to help elucidate the cardioprotective effects of SGLT2is observed in human clinical trials by summarizing the cardioprotective effects studied in SGLT2i animal models in the hopes that this may aid future clinical research of SGLT2is and its usage as a form of cardiac therapy.

## Rodent Models

The most utilized model currently to study the cardioprotective effects of SGLT2is are the rodent models which have also proven popular in T2DM studies [15]. This is due to the fact that there is a wide variety of rodent models to choose from, each with their specific advantages and disadvantages. Use of mice in rodent model studies of different diseases are often the first choice among rodents since mice are small in size, generally cost less to maintain and because the tools to genetically manipulate their genomes have been present since the 1980s, so they are more available and well understood [16]. Rats are also popularly used especially in cardio-specific and behavioral studies [17, 18]. Other popular murine species include gerbils, hamsters, and guinea pigs [19]. Thus, different rodent species have been used to study the various cardioprotective effects of SGLT2is.

### Cardiac Remodeling Effects

A major cardioprotective effect observed in rodent model studies of SGLT2is is cardiac remodeling effects which pertain to the improvement in cardiac function or rescue of cardiac cells in HF cases. In one of the very first rodent model studies conducted by Byrne et al. (2017) [20] to investigate the cardioprotective effects of SGLT2is, male C57Bl/6 nondiabetic mice were employed. To develop HF, animals underwent either sham or transverse aortic constriction surgery. As a post-surgical procedure, mice with induced HF were exposed to either a control vehicle or SGLT2i EMPA for 14 days. At the endpoint, cardiac function was assessed in vivo. While control HF mice underwent a progressive deterioration of cardiac function along the 14-day exposure time, this impact was blunted in the EMPA-treated group. An allocation to EMPA improved cardiac systolic function but did not significantly alter cardiac remodeling or diastolic function. Furthermore, in order to determine if the protective effects observed in the EMPA-treated mice with HF were due to other interfering factors controlling cardiac function (such as hemodynamics or ketone oxidation), authors assessed the functional state of vehicle- and EMPA-treated mice in an isolated perfused working heart. Despite matching pre-load and after-load pressures, identical insulin, fatty acid, and glucose concentrations, as well as absence of ketones, ex vivo perfused hearts still demonstrated significant improvements without any differences in heart rate in ex vivo cardiac output and cardiac work. This suggested potential of EMPA in delivering a continuous benefit in the secluded hearts, suggesting that the demonstrated effect in alleviating HF is a result of EMPA, ruling out any other blood-based environmental milieu linked to HF. This study being one of the first animal model studies of cardioprotective effects of SGLT2is clearly highlights the importance of post-translational studies required to study the intrinsic factors associated with SGLT2is and their role in cardiac remodeling. Further studies confirmed the importance of investigating potential pathways influencing these intrinsic factors of cardiac remodeling as a consequence of SGLT2is. As a result, the following studies that investigated SGLT2is’ cardiac remodeling effects in rodent models have also examined and suggested potential mechanisms and pathways involved including anti-metabolic and inflammatory pathways, oxidative stress mechanisms, and possible microbiota involvement.

In a study using T2DM KK-Ay mice, Li et al. (2019) [21] investigated the effect of SGLTi EMPA on myocardial injury (MI) of the left ventricle (LV) and the potential anti-inflammatory mechanism behind observed cardiac remodeling effects. Their investigation relied mainly on the gene expression analysis of TGF-β/Smad and Nrf2/ARE signaling pathways through histological and immunohistochemical analysis and western blotting techniques [21]. It was concluded that EMPA inhibits myocardial fibrosis partly through the inhibition of collagen formation and deposition via the classical transforming growth factor-β (TGF-β) and downstream Smad pathway and decreases oxidative stress via promoting nuclear erythroid 2-related factor 2 (Nrf2) translocation to the nucleus and activating Nrf2/antioxidant response element (ARE) signaling in the T2DM KK-Ay mice model [22]. Both pathways have been extensively studied for their involvement in tissue fibrosis [23] and oxidative stress management promoting collagen production [24]. Therefore, through this proposed mechanism, EMPA treatment was able to rescue the LV structure and function in T2DM mice. In a similar study also focusing on the cardiac remodeling benefits of SGLT2i and potential anti-inflammatory mechanisms, Penning et al. (2019) [25] studied whether glucose lowering induced by SGLT2i EMPA accelerates atherosclerosis regression using male C57BL/6 J mice induced with severe hypercholesterolemia and atherosclerosis progression [26]. Following induction of atherosclerosis progression, animals were injected with streptozotocin (STZ) to induce diabetes. Studied groups included a baseline group that was sacrificed after it demonstrated considerable atherosclerosis at the root of the aorta and another group where mice were subjected to a chow diet and low-density lipoprotein receptor (LDLR) sense oligonucleotides treatment to enhance the regression of atherosclerosis. This group was then subdivided into a control group and an EMPA-treated group, where treatment took over 21 days. In summary, their findings indicated that the mice treated with EMPA had significantly smaller atherosclerotic plaques, lower lipid levels, higher collagen levels, and an accumulation of CD68 + macrophages believed to contribute significantly to atherosclerosis progression [27]. According to these results, glucose reduction may accelerate atherosclerosis regression, possibly by reducing intra-plaque macrophage proliferation and decreasing leukocyte recruitment to vessel walls. However, additional research work is required to investigate which mechanism decreases glucose to mediate these effects in vivo*.*

In an alternative study looking into relating SGLT2i’s cardiac remodeling effects and several potential causative pathways, Lee et al. (2019) [28] used a spontaneous hypertensive rat (SHR) model to study the effect of SGLT2i EMPA and subsequent cardiac remodeling effects. Their findings showed that EMPA exhibited cardiac protection effects via improved atrial and ventricular remodeling and renal protection through significant reduction in creatinine levels, while plasma glucose levels were not affected. Aside from normalizing the end-systolic and end-diastolic volumes in SHR, TEMPA also normalized parameters assessed by echocardiography. Of importance, using histological analysis, there was a significant reduction in cardiac fibrosis in both atrial and ventricular tissues after treatment with EMPA. Moreover, the upregulation of atrial and ventricular expression of peroxisome proliferator-activated receptor-α (PPARα), acyl-coenzyme A dehydrogenase medium chain (ACADM), natriuretic peptide precursor A and B (NPPA and NPPB), and tumor necrosis factor-α (TNFα) was restored in SHR. Upregulation of these genes is significant as they are involved in a multitude of different pathways that could potentially lead to cardiac remodeling effects. PPARα and ACADM are both involved in fatty acid oxidation and metabolism [29, 30], expressions of both NPPA and NPPB encoding for ANP (atrial natriuretic protein) and BNP (brain natriuretic protein), respectively, have been shown to have blood pressure-lowering effects [31] as well as contribute to cardio-renal homeostasis [32, 33], and TNFα is a potent paracrine and endocrine mediator of inflammatory and immune functions that is responsible for mediating signaling pathways that play an important role, both in homeostasis and pathophysiology [34, 35]. The aforementioned genes show promising potential for research and are all subject to further studies to conclude their role in the cardiac remodeling effects of SGLT2is.

Lastly, in an unconventional study exploring the cardiac remodeling effects of an SGLT2i using T2DM mice, Lee et al. (2018) [36] studied whether SGLT2i DAPA improves generalized vascular dysfunction in T2DM mice with a secondary aim of determining the effects of DAPA on the gut microbiota. DAPA treatment for 8 weeks significantly reduced arterial stiffness in T2DM mice, as well as improved endothelial dysfunction and vascular smooth muscle dysfunction. These improvements on the vascular level were associated with alleviation of hyperglycemia and reduction in the inflammatory reaction. These findings came consistent with previous articles. It was demonstrated that DAPA has an effect of changing the microbial diversity in animals with diabetes. T2DM mice treated with DAPA showed specific taxa changes, but control mice did not, although their relevance to treatment efficacy has not been determined. SGLT2i treatment may provide cardiac remodeling benefits by improving generalized cardiovascular function, as the gut microbiome may play an important role in this process. However, the causative effect of alterations in the gut microbiota leading to cardiac remodeling benefits remains unclear, but this study presents a unique and alternative pathway of SGLT2i’s cardiac remodeling effects to those suggested previously [36].

### Ionic Remodeling Effects

In the following section, the rodent model studies that were used to focus on the relation between SGLT2i’s cardioprotective benefits and ionic remodeling effects will be discussed. In a combined rodent model study conducted by Chung et al. (2020) [37], Langendorff-perfused hearts isolated from mice, rats, and guinea pigs were used to study the effect of SGLTi EMPA on sodium/hydrogen exchanger-1 (NHE1) activity. The use of Langendorff-perfused hearts was required to conduct preliminary data to test whether cardioprotective actions may become apparent only in the intact beating heart compared to cardiomyocytes. Data extracted from the mentioned study demonstrated that SGLT2i, EMPA, DAPA, and CANA failed to inhibit NHE1 (measured in terms of initial [Na^+^] concentration and pH levels) in isolated cardiomyocytes or Langendorff-perfused beating hearts. These results were consistent across all three species used (mice, rats, and guinea pigs), excluding a possible species-dependent response to SGLT2i. This study proposes eliminating the involvement of attenuated NHE1 activity and intracellular [Na^+^] concentration as possible ionic remodeling effects of SGLT2i’s cardioprotective benefits within rodent models. Other rodent studies focused on other potential ionic remodeling mechanisms. In a study conducted by Durak et al. (2018) [38], rats were fed a high-carbohydrate diet for 28 weeks to induce metabolic syndrome (MetS)-stimulated cardiac dysfunction followed by treatment with either DAPA, insulin (INSU), or control vehicle for 2 weeks. MetS rats that were exposed to DAPA treatment demonstrated a substantial increase in blood pressure, low heart rate with depressed left ventricular function, and relaxation of the aorta and prolonged Q–R interval. Prolonged-action potentials were preserved in DAPA-treated groups, in a more notable manner than in the group with INSU-treatment, through amplifying depressed voltage-gated K^+^-channel currents. In contrast to INSU-treatment, DAPA preserved the depolarized mitochondrial membrane potential, as well as significantly enhanced cytosolic Ca^2+^-homeostasis. Furthermore, in cardiomyocytes obtained from MetS rats, DAPA induced a significant increase in voltage-gated Na^+^-currents and intracellular pH, as well as increased levels of protein thiol oxidation, and ADP/ATP ratios. Moreover, DAPA treatment normalized the increases in the mRNA level of SGLT2 in MetS rat hearts. Based on these findings, conclusions can be made on the involvement of SGLT2i in augmenting mitochondrial function and oxidative stress via the improvement of fusion–fission proteins through its glucose-lowering effect, leading to ionic homeostasis of Ca^2+^ and Na^+^ in cardiac cells by binding to cardiac SGLT2 as evident in increased mRNA levels of SGLT2 in cardiac cells. This proposed ionic remodeling pathway proves to be highly promising in explaining SGLT2i’s cardioprotective benefits but requires further study over a longer duration to understand the longevity of the beneficial treatment.

### Metabolic Remodeling Effects

In this review, the final section of the rodent model studies of SGLT2i’s cardioprotective benefits tackles the popular metabolic remodeling “thrifty fuel” hypothesis. The “thrifty fuel” hypothesis refers to the utilization of ketone metabolism by cardiac cells due to the hindered availability of glucose as a result of SGLT2 inhibition, hence undergoing a metabolic remodeling effect [39]. In a study conducted by Nambu et al. (2020) [40], the effects of SGLT2i EMPA were investigated on exercise endurance plus the function of skeletal muscle mitochondria with the oxidation process of fatty acid in male C57BL/6 J mice model with HF after the induction of MI and administration of EMPA. It was observed that SGLT2i improved exercise endurance capacity in HF mice, without affecting cardiac functions post-MI, among several novel findings regarding the effects of SGLT2i on skeletal muscle abnormalities associated with HF. It has been shown that EMPA improves endurance capacity by improving mitochondrial fatty acid oxidation in HF mice. This came in line with previous studies demonstrating lipolysis in adipose fat tissues of mice subjected to SGLT2i [41, 42]. The mechanism proposed by the researchers suggests that the reduction in fat weight is to some extent dependent on increased energy expenditure or improved fatty acid oxidation, yet, no molecular evidence via enhanced 5′ adenosine monophosphate-activated protein kinase (AMPK) expression [43] and increased *β*-hydroxybutyrate (β-OHB) levels [44] were found. Together, these results propose that increased β-OHB levels in response to EMPA administration may trigger the hyperacetylation of fatty acid β-oxidation enzymes, leading to the enhancement of fatty acid oxidation and increased β-OHB levels in skeletal muscle; however, more molecular markers need to be investigated to prove this hypothesis. In a similar study conducted by Oshima et al. (2019) [45] also focusing on β-OHB levels had a similar conclusion. Using T2DM Otsuka Long-Evans Tokushima Fatty (OLETF) rats and Long-Evans Tokushima Otsuka (LETO) control rats induced with MI, they were able to study the effects of SGLT2i EMPA on the acute survival rate post-MI and the possible modification of cardiac metabolomes and antioxidative proteins. Their findings concluded that treatment with EMPA led to blood and myocardial β-OHB levels elevation and an enhanced level of β-OHB was accompanied by preservation of ATP level after MI in tissue. These data proposed that higher delivery of ketone bodies to the heart with ventricular dysfunction is advantageous in terms of energy metabolism even if the protein expression of genes that regulate ketone oxidation and transport such as monocarboxylate transporter 1 (MCT1), D-beta-hydroxybutyrate dehydrogenase-1 (BDH1), and succinyl-CoA:3-oxoacid CoA transferase (SCOT) are not upregulated in the myocardium. Lack of upregulated gene expression could be due to duration and severity of the MI as well as the use of T2DM rat models as previous studies investigating ketone utilization post-HF reported upregulation and utilization of ketone oxidation proteins [46–49]. The lack of direct molecular evidence in both previously discussed studies [40, 45] highlights the need for post-translational investigations that may provide more novel insights into the cardioprotective mechanism of SGLT2is and their direct interference with protein and genetic profiles of metabolic remodeling effects.

## Pig Models

Pigs are considered more suitable for human research than rodents, particularly regarding physiological factors and biomedical applications [50]. Pigs have been used in past studies to investigate several areas linked to human nutritional sciences [51, 52], neuroscience [53, 54], and maternal and progeny interactions [55]. Moreover, sequencing the pig genome has aided employing the pig as a model for human studies through unveiling the genetic similarities and differences between pigs and humans [56]. Due to the pig model’s suitability to elucidate metabolic queries of the human metabolism, the few SGLT2i pig model studies revolve mainly around metabolic alterations following MI and SGLT2i treatment with a focus on the cardiac remodeling of swine hearts. The first of these studies was conducted by Baker et al. (2019) [57] in which the study aimed to investigate the impact of SGLT2i CANA on cardiac contractile function, substrate utilization, and efficiency before and during regional MI in normal, metabolically healthy swine. Initial findings of the study detected mRNA expression of both SGLT1 and SGLT2 in swine hearts and kidneys at a ratio of 1:100. Further data from this study demonstrates that CANA preserves cardiac contractile function and efficiency during acute regional MI through acute effects on cardiac volume regulation that cannot be explained by myocardial fuel switching also known as the “thrifty fuel” hypothesis as no changes in myocardial uptake of glucose, lactate, ketones, or free fatty acid were seen in this study; however, since metabolically normal pigs were used, this could have limited the examination of the metabolic remodeling effects. Furthermore, the study suggests considering the involvement of NHE-1 inhibitory activity to explain cardiac improvements of SGLT2i as subsequent improvement in cytosolic Ca^2+^ handling produces a myriad of beneficial effects on regulatory and contractile proteins within cardiac cells [58]. Further investigations are required to investigate both possible mechanisms for improvements observed in cardiac function during MI in pig models, with an emphasis on using metabolically disordered models within future studies. As if in response, both Zhang et al. (2019) [59] and Santos-Gallego et al. (2019) [60] used metabolically disordered and nondiabetic pig models, respectively, to further investigate cardioprotective effects of SGLT2i. In the study conducted by Zhang et al. (2019) [59], the findings helped build a more intensive understanding of the structural remodeling of the heart, heart function and sympathetic tone variations, and the subsequent molecular mechanism involved in HF cases associated with SGLT2i DAPA within nondiabetic pig models. This was evidenced by the decrease in both systolic (SBP) and diastolic (DBP) blood pressure and preventative progression of LV concentric hypertrophy cardiac remodeling and left atrium (LA) dilation. In addition, using echocardiography and an invasive hemodynamic assessment, no discernible effects of 9 weeks of DAPA treatment on LV fibrosis or diastolic function were observed in HF pigs. Furthermore, DAPA treatment lowered plasma adrenal medullary hormone levels substantially and strongly alleviated the sympathetic tension in the aorta. In the non-treated HF pigs, the inflammatory response and the NO-cGMP-PKG (nitric oxide-cGMP-dependent protein kinase) signaling pathway were deteriorated, and both were improved by DAPA treatment. This is critical as the NO-cGMP-PKG signaling pathway is a vital pathway for vascular dilation leading to the decrease of blood pressure by decreasing Ca^2+^ sensitization and/or activating Ca^2+^-activated K^+^ channels to reduce the intracellular Ca^2+^ concentration [61, 62]. This proposes another potential pathway explaining both cardiac and ionic remodeling effects observed in SGLT2i cardioprotective benefits that should be further explored. Alternatively, in the study conducted by Santos-Gallego et al. (2019) [60] using nondiabetic pig specimens, data demonstrated that using SGLT2i EMPA to inhibit SGLT2 chronically improves adverse anatomical LV remodeling, enhances LV systolic function, and decreases neurohormonal activation in a nondiabetic pig model of HF. EMPA appears to have cardioprotective effects through the shifting in myocardial fuel metabolism away from glucose towards cardiac utilization of ketone bodies (KB), free fatty acids (FFA), and branched-chain amino acids (BCAA), which improves myocardial energetics and cardiac function. This was confirmed via uptake levels of respective metabolites in the myocardium as well as increased protein expression and activity of metabolic enzymes SCOT, BDH1, and lactate dehydrogenase (LDH), providing more evidence towards the “thrifty fuel” hypothesis.

## Rabbit Models

Rabbit models act as an intermediate animal model between rodents and pigs due to their moderate size. Rabbits offer several potential advantages over other species such as having operable yet smaller and cheaper hearts than pigs. Physiologically, rabbit cardiac structures are more similar to human hearts compared to rodents, especially in cellular electrophysiology and Ca^2+^ transport [63]. This is crucial as modifications in ion channel and Ca^2+^ transporter function or expression contribute directly to impaired contractility and arrhythmias, prompting HF [64]. Other advantages of rabbit models include their docile and non-aggressive nature, inexpensive cost, having shorter vital cycles (gestation, lactation, and puberty) than larger animals, and easier handling, observability, and breeding. Disadvantages of rabbit models include lack of available rabbit-specific facilities, operative medicines, and experimental literature [65].

Similar to pig models, there is not much research carried out on SGLT2i’s cardioprotective effects using rabbits. The few studies published focus on the effects of SGLT2i on [Na^+^] and [Ca^2+^] cardiac concentrations and SGLT2i’s anti-atherosclerotic and anti-inflammatory effects. In a study conducted by Baartscheer et al. (2017) [66], an increase in the concentration of extracellular glucose, from 5.5 to 11 mmol/l, resulted in increased cytoplasmic [Na^+^] and cytoplasmic [Ca^2+^] levels in rabbit cardiomyocytes, simulating diabetic conditions in cardiac cells. By using SGLT2i EMPA treatment, NHE flux was directly inhibited, causing a reduction in cytoplasmic [Na^+^] and cytoplasmic [Ca^2+^] and increased mitochondrial [Ca^2+^] in rabbit cardiomyocytes. After pretreatment with the NHE inhibitor, Cariporide, these effects of EMPA were strongly reduced. EMPA also affected cytoplasmic [Na^+^] and NHE flux in the absence of extracellular glucose. These results indicate a clear relationship between SGLT2i’s presence and the reduction of cardiac cytoplasmic [Na^+^] and [Ca^2+^] concentrations resulting in the improvement of heart arrhythmia, oxidative stress, and heart failure. This study compounded with previous studies of SGLT2i using pig [51] and rodent [38] models provides further evidence of the involvement of [Na^+^] and [Ca^2+^] concentrations in ionic remodeling effects associated with the cardioprotective benefits of SGLT2i as alterations in ion channel and Ca^2+^transporter function or expression are thought to contribute directly to depressed contractile performance and arrhythmogenesis [67, 68]. Alternatively, another SGLT2i study involving rabbit models looked into the anti-atherosclerotic and anti-inflammatory effects of SGLT2i. Lee et al. (2020) [69] studied the changes in the polarization of M1 and M2 macrophages and the expression of a number of inflammatory mediators as effective elements in the protective mechanism of SGLT2i DAPA against the progression of atherosclerosis in a normoglycemic atherosclerotic rabbit model. The results showed that treatment with DAPA reduces development of atherosclerotic lesions in the normoglycemic rabbit model. Lipid accumulation, intimal proliferation, and pro-inflammatory marker levels were all suppressed by SGLT2i treatment, suggesting that the drug exerts multiple beneficial anti-atherosclerotic effects.

## Zebrafish Models

Among vertebrate animal models, zebrafish have several advantages for cardiovascular disease investigations. Zebrafish embryos develop fast, and in only a few days, major organs including the heart become fully functional. In addition, zebrafish embryos can easily be imaged because of their transparent skin, enabling direct visualization of the cardiovascular system. Zebrafish share similar cardiac physiology and structure to human hearts. One other advantage of zebrafish research is the availability of the reverse genetic approaches such as morpholino antisense oligos [70]. Currently, only one zebrafish animal model study of SGLT2i’s cardioprotective effects has been conducted. In the study, Shi et al. (2017) [71] examined biomarker changes elicited by SGLT2i EMPA in a validated zebrafish model of heart failure. Their results support the idea that SGLT2 inhibition is an effective approach to control aberrant cardiac remodeling and mortality, in part through modulating BNP and ANP signaling pathways. The increase in NPPA (ANP) and NPPB (BNP) expression in zebrafish embryos that were exposed to aristolochic acid (AA) to induce HF was reversed in HF embryos treated with EMPA. Similar analyses demonstrated that EMPA alleviated the increase of the expression of the pro-inflammatory genes cox-2 (cyclooxygenase-2) and IL-1β (interleukin-1β) upon AA stimulation. To further confirm if the effects of EMPA were mediated through SGLT2 inhibition, SGLT2 (*slc5a2* gene) morpholino knockdown (MO) zebrafish embryos were created and treated with AA to see if similar results to the EMPA-treated embryos could be produced. Their findings showed that the SGLT2 MO zebrafish models have similar upregulated expressions of NPPB as well as reduced the expression of anti-inflammatory genes cox-2 and IL-1β. EMPA-REG Outcome data reveal the first translational and mechanistic explanations for dramatic and precocious outcomes and clearly links cardioprotective benefits of SGLT2i with inhibition of SGLT2. Based on this study, investigation of downstream affecters of SGLT2i inhibition should be investigated.

## Next Steps: Limitations and Future Directions

The key studies previously mentioned in this review studying SGLT2i’s cardioprotective effects in animal models have highlighted potential limitations associated with various animal models that should be considered in future directions. A criteria matrix of the advantages and limitations of the animal models mentioned in this review can be found in Table 2.

**Table 2:**
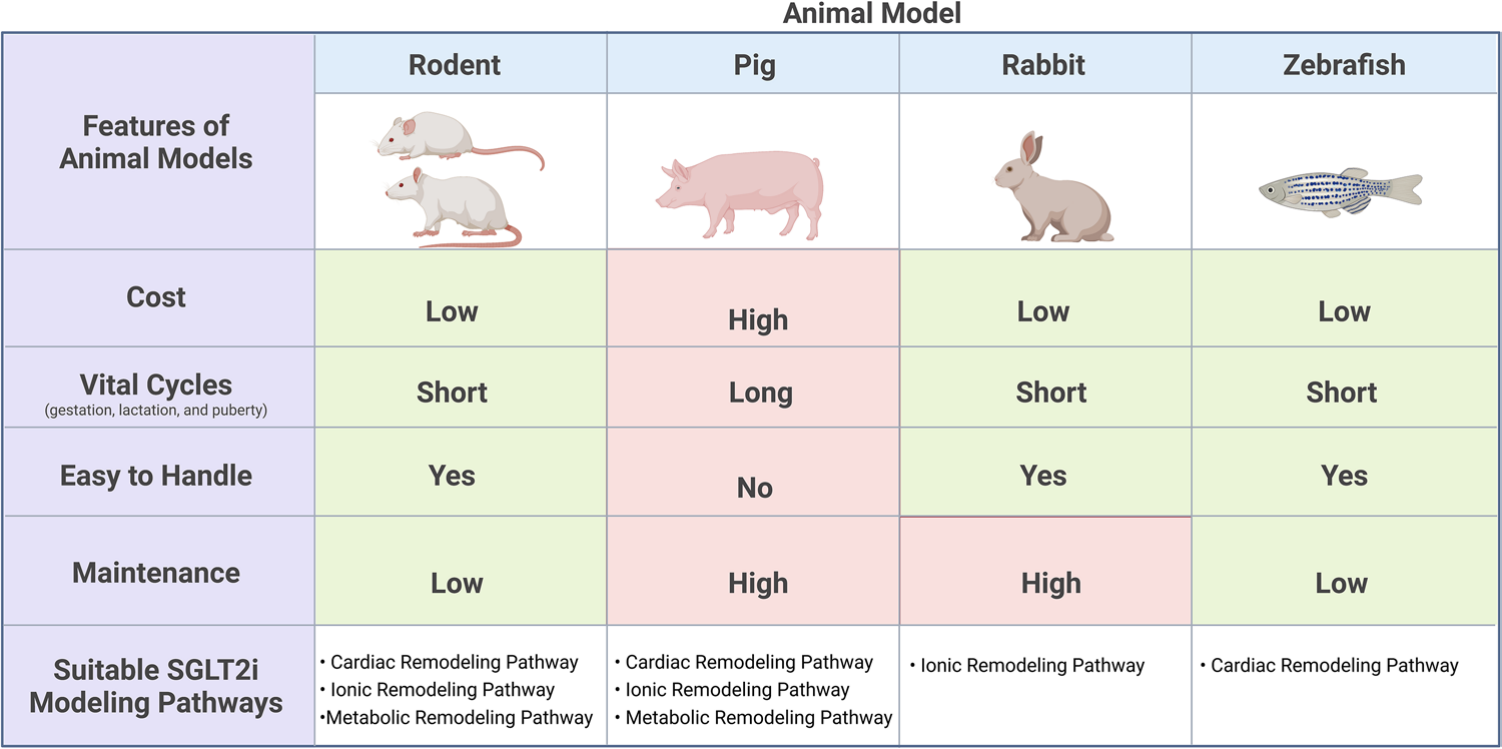
Animal model criteria matrix with suggested suitable SGL2i modeling pathways. Created with Biorender.com

### Limitations

As previously mentioned in this review, the most popular animal model to study the cardioprotective effects of SGLT2is is the rodent model. This is mostly due to convenience since they are cheaper, smaller, are easily bred, have short vital cycles, and are genetically modifiable [16]. However, in regard to their suitability as an animal model for cardiac studies, several critical differences between humans and rodents limit their potential as an ideal model for elucidating the pathogenesis of cardioprotective effects of SGLT2is [72]. These limitations include the fact that rodents do not naturally develop T2DM and are resilient to T2DM, cardiac dysfunction, and obesity induced via diet; therefore, this poses a problem in studies of the cardioprotective effects of SGLT2i in rodents as they are arguably not a reliable translational model [73]. In addition, rodents are also nocturnal animals which differs with human lifestyle and behavior [73]. Rodent models also have limitations due to their small size, and they do not go through a naturally occurring menstrual phase, both of which are shared with rabbits and zebrafish [74]. On the other hand, pigs do undergo an estrous cycle, similar to a menstrual cycle in humans, and are genetically the most similar of the animal models mentioned in this review to humans [56, 75]. However, similar to rabbits, pigs are considered expensive mainly due to the specific housing facilities and operative medicines required for experimentation. Lastly, zebrafish have similar advantages to rodents in regard to their inexpensive cost, small size, short gestation and growth period, genetic modifiability, and easy breeding tactics; however, they require special aquatic facilities that are highly sensitive to changes in pH and temperature as well as complicating certain cardiac-specific assays, such as EKG measurements and drug administration. Zebrafish differ morphologically from humans and have a relatively smaller library of zebrafish-specific antibodies for immunohistochemistry studies, and although genetic modification is possible with zebrafish, they are hindered by long mutant generation timelines, taking approximately 4–6 months to generate one transgenic line [76].

### Future Directions

A major requirement for future animal models is the utilization of intact hearts, whether in vivo or using perfused hearts, rather than the use of cardiomyocyte cell lines as they present a clearer image of various pathways linked to SGLT2i’s cardioprotective effects as well as providing more insight into potential post-translational properties [37]. Another trend followed by many of the studies mentioned is the use of nondiabetic HF models to study whether cardioprotective effects of SGLT2is are an isolated phenomenon or are linked to blood glucose improvements via T2DM treatment, and this should be continued. Utilization of nondiabetic HF models in parallel with T2DM models or as the primary model within the study is crucial moving forward as it will further elucidate the role of SGLT2is and their observed cardiac improvements.

Furthermore, it is worth looking into new animal models that are more closely related to humans such as non-human primates (NHPs). NHPs provide an exceptional advantage over any other animal model due to their similarities to humans, genetically and physiologically, while still being easily manipulable with regard to diet and drug admission [73, 77]. However, NHPs are considerably more expensive, require highly specialized housing facilities and expertise, and present further ethical concerns [77]. Within the scope of SGLT2i research, a comparative study conducted by Zhang et al. (2019) analyzed the metabolites excreted by humans, monkeys, and rats following SGLT2i treatment [78]. The results found that metabolism by rats is oxidation-dominant, whereas metabolism by monkeys is glucuronidation-dominant, resembling metabolism patterns in humans [78]. In addition, all metabolites detected in monkeys were also found in humans, further confirming that NHPs provide a more realistic animal model for SGLT2i studies [78].

Another animal model worth looking into are Drosophilae, or more commonly known as fruit flies, due to their inexpensive cost, even for a high number of specimens [79]. Drosophilae are also fairly easy to manage and have been extensively studied, having been previously used in T2DM and insulin signaling studies, and could pose as a potential animal model for the study of SGLT2i’s cardioprotective effects [80].

Lastly, further translational studies are required to understand the mechanistic consequences of SGLT2is. Upstream and downstream proteins or metabolites within pathways of interest should be targeted in future studies to help elucidate SGLT2i’s exact cardiac involvement and to aid in creating a full comprehensive understanding of SGLT2i’s cardioprotective mechanism.

## Conclusion

In summary, the study of SGLT2i’s cardioprotective effects has been explored through the usage of various animal models ranging from rodent, pig, rabbit, and zebrafish models. Collectively, the studies suggest specific pathways of interest that should be further investigated to clarify how SGLT2is are involved in observed cardiac improvements [8–11, 13]. The cardioprotective effects of SGLT2i can be classified into three separate remodeling outcomes: cardiac remodeling, ionic remodeling, and metabolic remodeling. Cardiac remodeling is a result of improved cardiac tissue plasticity, elasticity, and overall function that can be attributed to anti-inflammatory properties of SGLT2i. As a result, several anti-inflammatory pathways and associated proteins have been proposed to be involved in the cardioprotective mechanism of SGLT2i which include TGF-β, SMAD, Nrf-2, ARE, PPARα, ACADM, NPPA(ANP), NPPB(BNP), TNFα, cox-2, IL-1β, IL-16, NO-cGMP-PKG, macrophage proliferation and leukocyte recruitment, and gut microbiome involvement (Table 2). Ionic remodeling is a result of improved ionic homeostasis within cardiac tissues following SGLT2i treatment. As a result, several pathways and proteins associated with ionic homeostasis in cardiac tissues have been proposed to be involved in the cardioprotective mechanism of SGLT2i which include mitochondrial Ca^2+^ and cytoplasmic Na^+^ homeostasis, NHE-1, Mfn-1, Mfn-2, and Fis-1 (Table 2). Metabolic remodeling is a result of myocardial fuel metabolism shifting from glucose to ketones, improving myocardial energy production. This would be a result of the lack of bioavailable glucose following SGLT2i treatment. Proposed mechanisms and proteins include AMPK, β-OHB, and associated metabolic enzymes, MCT1, BDH1, SCOT, and LDH (Table 2). The identification of these potentially involved pathways and proteins associated with SGLT2i’s cardioprotective effects by the extensive research done using animal models have paved the way for more breakthrough findings in regard to SGLT2i’s and observed cardiac improvements (Table 3).

**Table 3:**
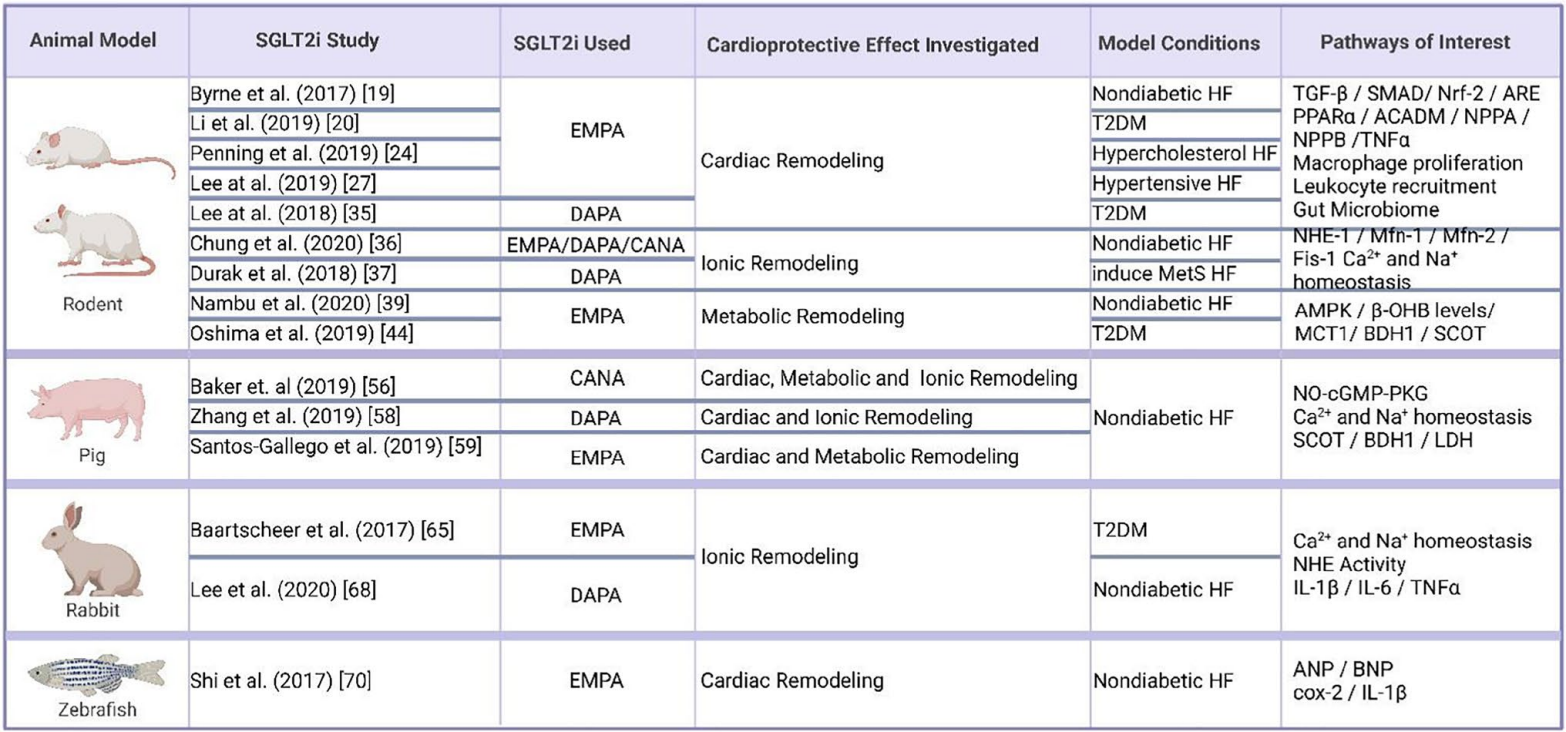
Summary table of SGLT2i animal model studies. Created with Biorender.com

## Data Availability

All data supporting the findings of this review are available within the paper.

## Declarations

### Disclaimer

The funders had no role in the study’s design; the collection, analyses, and interpretation of data; the writing of the manuscript; or in the decision to publish the results.
